# Effects of Acute and Chronic Hyperglycemia on Neurotrophic Factors in the Gut: GDNF and NT3 Expression in Myenteric Ganglia

**DOI:** 10.3390/biomedicines14102249

**Published:** 2026-10-04

**Authors:** Zita Szalai, Benita Onhausz, Mária Bagyánszki, Titanilla Szögi, Nikolett Bódi

**Affiliations:** 1 Department of Physiology, Anatomy and Neuroscience, Faculty of Science and Informatics, University of Szeged, Közép Fasor 52, H-6726 Szeged, Hungary; zszalai@bio.u-szeged.hu (Z.S.); onhausz.benita@bio.u-szeged.hu (B.O.); bodi.nikolett@bio.u-szeged.hu (N.B.); 2 Department of Pathology, Faculty of Medicine, University of Szeged, Állomás u. 1, H-6725 Szeged, Hungary; szilagyi-szogi.titanilla.zita@med.u-szeged.hu

**Keywords:** hyperglycemia, glial cell line-derived neurotrophic factor, neurotrophin-3, myenteric neurons, diabetic neuropathy, type 1 diabetes

## Abstract

**Background/Objectives**: Glial cell line-derived neurotrophic factor (GDNF) and neurotrophin-3 (NT3) are essential neurotrophic factors that continue to be expressed in the mature intestine. Myenteric neurons are damaged by hyperglycemia to varying degrees in the different gut segments. Therefore, our aims were to identify the gut-segment-specific expression patterns of GDNF and NT3 in acute and chronic hyperglycemic rat models. **Methods**: Segments of the duodenum, ileum, and colon were dissected one week (acute experiment) or ten weeks (chronic experiment) after the streptozotocin-induced hyperglycemia. GDNF–HuC/HuD fluorescent immunohistochemistry, NT3 enzyme-linked immunosorbent assay, and NT3 immunogold electron microscopy were performed in rat myenteric ganglia. **Results**: The proportion of GDNF–immunoreactive myenteric neurons did not change in gut segments in acute hyperglycemia. However, during chronic diabetes, it was elevated in the colon and remained unchanged in the duodenum and ileum. Expressional alterations of NT3 were investigated in the small intestine. In duodenal myenteric ganglia, acute hyperglycemia induced a significant increase in the number of NT3-labeling gold particles, and its density was markedly increased in chronic diabetes, which was partially prevented by insulin treatment. In the ileum, NT3 density increased in acute hyperglycemic rats, while it decreased in chronic diabetic rats. **Conclusions**: Induction of neurotrophic factors was specific to different gut segments during acute and chronic hyperglycemia. These results suggest that increased colonic GDNF and duodenal NT3 expression may reflect a compensatory response of myenteric neurons to hyperglycemic states.

## 1. Introduction

As a complication of chronic hyperglycemia, diabetic patients often suffer from gastrointestinal dysfunctions such as delayed gastric emptying, diarrhea, abdominal pain, or constipation. The enteric nervous system (ENS) is also affected by diabetic neuropathy, which contributes to the pathogenesis of intestinal motility dysfunction [1]. The enteric neurons and glial cells that make up the ENS are grouped in ganglia, which are organized into plexuses. The myenteric plexus of the ENS is located between the longitudinal and circular muscle layers and regulates the motor functions of the gut [2]. The diabetic damage to enteric neurons is thoroughly studied, but that of enteric glial cells is less well known. We previously described gut segment-specific enteric neuropathy in a streptozotocin (STZ)-induced diabetic rat model (the same model was used in this study). In the duodenum, the total neuronal number did not change, while distally the neuronal loss was increasingly pronounced; it was most severe in the colon. Moreover, the alterations in gut motility were in relation to these changes in cell number. The insulin treatment was ineffective in some pathological aspects and gut regions [3,4]. Therefore, it is important to understand the pathological mechanisms underlying the damage to myenteric ganglia by hyperglycemia to improve the quality of life of diabetic patients.

Glial cell line-derived neurotrophic factor (GDNF) and neurotrophin-3 (NT3) are essential neurotrophic factors with pleiotropic effects in the development of the neural crest-derived ENS [5,6]. They also participate in the regulation of neuronal differentiation and synaptic plasticity [7,8]. The literature is more abundant regarding their roles in embryonic development; however, increasing evidence suggests their importance in different diseases in adulthood. The GDNF-induced signaling is mediated mainly via a multi-component receptor system consisting of the GDNF family receptor alpha 1 (GFRα1) and phosphorylated rearranged during transfection (RET) receptor tyrosine kinase [9,10]. Moore et al.’s [6] study on the relevance of GDNF has shown that GDNF-deficient mice did not develop an ENS. Moreover, GDNF plays an important role in the structural and functional postnatal development of myenteric neurons [8]. Virtanen et al. [11] described the relationship between interindividual differences in GDNF levels and some normally occurring interindividual variations in the size, composition, and function of ENS in humans and mice. Contrary to its name, GDNF is expressed in many cell types. It is mainly expressed by neurons in the adult healthy brain, but it is expressed de novo by glial cells—microglia, astrocytes—in diseases with neuroinflammation [12,13,14]. In the gut wall, smooth muscle layers are the main source of GDNF [15]. The highest GDNF mRNA level is found in the circular layer, while GDNF mRNA expression is significantly lower in the myenteric plexus [16]. Enterocytes also contribute to GDNF synthesis; furthermore, GDNF contributes to intestinal epithelial wound healing and barrier maturation [17]. Enteric glial cells are the major source of the increased amount of GDNF in the inflamed gut [18,19] and after acute ischemia–reperfusion injury [20]. The protective role of GDNF in hyperglycemia has been verified in some studies. It has been demonstrated that GDNF reversed hyperglycemia-induced apoptosis of enteric neurons both in enteric neuronal cell culture and in a glial fibrillary acidic protein–GDNF transgenic diabetic mouse model. Moreover, GDNF overexpression prevented the loss of the nitrergic subpopulation of enteric neurons and improved intestinal motility compared to wild-type mice [21]. Luo et al. [22] have provided novel insight into the protective role of enteric glial cells via the release of neurotrophic factors in hyperglycemia.

NT3 is synthesized as a larger precursor form (proNT3) that is cleaved to yield the mature form [23]. Recent studies suggest that proNT3 is an active, signaling precursor [24]. ProNT3 and the mature form also activate a co-receptor complex; proNT3 utilizes p75 neurotrophin receptor (p75^NTR^) and sortilin, while the mature NT3 uses p75 ^NTR^ and tropomyosin receptor kinases (Trks). The cellular response to these complexes can be diverse and opposing, such as apoptosis or cell survival during neural development and pathophysiologic states [25]. It has recently been reported that proNT3, but not mature NT3, contributes to chemotherapy- or trauma-induced neuropathic pain in mice dorsal root ganglia [26,27]. Mature NT3 plays an essential role in the development of ENS, where it promotes the survival and differentiation of a subset of neurons and glia. In NT3 overexpressing transgenic mice, hyperplasia of myenteric ganglia was observed, but no changes in submucosal ganglia were found [7,28]. NT3 binds to TrkC with high affinity and interacts with TrkA and TrkB with lower affinity [29,30]. In TrkC knockout mice, a 30% loss of myenteric neurons was detected, and a more severe decrease was found in the submucosal ganglia [28]. In the intestinal wall, non-neuronal cell types have also been identified as a source of NT3, namely smooth muscle cells [31] and enteroendocrine cells [32]. Several pathological implications for NT3 have been described in diabetic neuropathy. In diabetic patients, the NT3 concentration of affected skin biopsies was higher than in controls [33]. NT3 treatment prevented mitochondrial dysfunction in sensory neurons of diabetic rats [34]. Intrathecal NT3 treatment of diabetic mice increased the abundance of dermal myelinated fibers and the number of Merkel cells associated with fibers [35].

Earlier findings demonstrated that the hyperglycemia-induced alterations in the ENS and its environment are intestinal segment-specific. The bidirectional crosstalk between different cell types of gut wall layers such as neurons, glia, immune, and enteroendocrine cells also has a regional character, which is influenced by microbiota [4,36,37,38,39]. The segment-specific pattern of myenteric neuronal loss raises the question of whether the neurotrophic factors can contribute to it in different gut regions. Therefore, our aims in this study were to provide a comprehensive, gut-segment-specific characterization of the expression of GDNF and NT3 neurotrophic factors in acute and chronic rat models of type 1 diabetes.

## 2. Materials and Methods

### 2.1. Animal Models

Adult male Wistar rats (Toxi-Coop Zrt., Balatonfüred, Hungary) were kept on standard laboratory chow (Farmer-Mix Kft., Zsámbék, Hungary) and had *ad libitum* access to tap water. All animal experiments were carried out in accordance with the National Institutes of Health (Bethesda, MD, USA) guidelines, and the EU Directive 2010/63/EU for the protection of animals used for scientific purposes was strictly followed. All of the experiments were approved by the National Scientific Ethical Committee on Animal Experimentation (National Competent Authority) with license numbers XX./1636/2019 and XX./1787/2024.

For the 1-week (acute) hyperglycemic experimental model, the rats (200–250 g) were divided randomly into two groups: acute hyperglycemic (aD, *n* = 6) and age-matched controls (aC, n = 5). For the 10-week (chronic) diabetic experimental model, the rats (180–250 g) were divided randomly into three groups: diabetics (D, *n* = 7), insulin-treated diabetics (ID, *n* = 8), and age-matched controls (C, *n* = 7). Randomization was performed using Microsoft Excel 2016. Hyperglycemia was induced by a single intraperitoneal injection of STZ (Sigma-Aldrich, Budapest, Hungary) at 60 mg/kg [3]. Controls were treated with vehicles (i.p. citrate buffer, pH 4.5). The animals were considered diabetic if the non-fasting blood glucose concentration was higher than 18 mmol/L. From this time on, the rats of the insulin-treated diabetic group received a subcutaneous injection of insulin (Humulin M3, Eli Lilly Nederland, Utrecht, The Netherlands) each morning (3 IU) and afternoon (4 IU). The chronic diabetic and control rats were dosed subcutaneously with equivalent volumes of saline. The blood glucose level and weight of rats were measured daily in the acute experiment, and these were monitored weekly in the chronic experiment (after the active nocturnal period, prior to insulin administration in the case of the ID group). Diabetic animals that recover spontaneously, or whose glucose level decreases to under 18 mmol/L during the experimental periods, were excluded from the study.

### 2.2. Tissue Handling

One or ten weeks after the onset of hyperglycemia, the animals were killed by cervical dislocation under deep chloral hydrate anesthesia (375 mg/kg i.p.). The gut segments from the proximal duodenum (1 cm distal to the pylorus), distal ileum (1 cm proximal to the ileo-cecal junction), and proximal colon of all experimental groups of rats were dissected and rinsed in a 0.05 M phosphate buffer (PB; pH 7.4). Samples were processed for fluorescent immunohistochemistry, enzyme-linked immunosorbent assay (ELISA), and quantitative immunogold electron microscopy. For double-labeling fluorescent immunohistochemistry, the intestinal segments were cut along the mesentery, pinched flat, and fixed overnight at 4 °C in a 4% buffered formaldehyde solution (Molar Chemicals, Halász-telek, Hungary). The samples were then washed, the mucosa, submucosa, and circular smooth muscle were removed, and whole-mounts with the myenteric plexus adhering to the longitudinal muscle were prepared. For the ELISA, the 3 cm-long gut segments were cut along the mesentery and pinched flat. After removing both the mucosa and submucosa, the layers of intestinal smooth muscle, including the myenteric plexus, were snap-frozen in liquid nitrogen and stored at −80 °C until use. For post-embedding electron microscopy, small pieces (2–3 mm) of the gut segments were fixed in a 2% paraformaldehyde and 2% glutaraldehyde solution and then further fixed for 1 h in 2% OsO_4_. After rinsing in buffer and dehydrating in increasing ethanol concentrations and acetone, they were embedded in Embed812 (Electron Microscopy Sciences, Hatfield, PA, USA).

### 2.3. Fluorescent Immunohistochemistry

For double-labeling fluorescent immunohistochemistry, whole-mount preparations derived from different gut segments were immunostained with primary antibodies against GDNF and HuC/HuD markers. Briefly, the whole-mounts were washed and permeabilized with 0.025% Triton X-100 in tris(hydroxymethyl)aminomethane-buffered saline (TBS). After blocking for 2 h with 1% bovine serum albumin (BSA) and 10% normal goat serum in TBS, the whole-mounts were incubated overnight with anti-GDNF and pan-neuronal anti-HuC/HuD at 4 °C (Table 1). After washing in TBS with 0.025% Triton X-100, sections were incubated with anti-mouse Cy^TM^3 and anti-rabbit Alexa Fluor 488 secondary antibodies (Table 1) for 1 h at room temperature, followed by washing in TBS and mounted on slides in Fluoromount^TM^ Aqueous Mounting Medium (Sigma-Aldrich, Budapest, Hungary). Negative controls were performed by omitting the primary antibodies, when no immunoreactivity was observed. Immunostained specimens were observed and photographed with a Zeiss Imager Z.2 fluorescent microscope equipped with an Axiocam 506 mono camera (Zeiss, Jena, Germany). A total of 30–50 myenteric ganglia were taken from each intestinal segment and experimental group, and the proportion of myenteric neurons that were immunoreactive for GDNF was counted per ganglion.

### 2.4. Tissue NT3 Concentration

Intestinal tissue samples, including the intestinal smooth muscle layers with the myenteric plexus, were frozen in liquid nitrogen, crushed into powder in a mortar, and homogenized in phosphate-buffered saline (PBS, pH 7.4). Tissue homogenates were centrifuged at 5000 rpm for 20 min at 4 °C. A rat NT3 ELISA kit (Cat. No. GA-E0551RT, GenAsia Biotech Co., Shanghai, China) was used to test the NT3 content of intestinal tissue samples. Quantitative ELISA was performed according to the manufacturer’s instructions. Optical density of each well was measured at a wavelength of 450 nm (Benchmark Microplate Reader; Bio-Rad, Budapest, Hungary). The tissue NT3 concentrations were expressed as ng/mg protein.

A commercial protein assay kit was used for the determination of total protein content in tissue samples. Bradford reagent was added to each sample. After mixing and following a 10 min incubation, the samples were assayed spectrophotometrically at 595 nm. The total protein level was expressed as mg protein/mL.

### 2.5. Quantitative Post-Embedding Immunohistochemistry

Embedded blocks originating from small intestinal segments were used to prepare ultrathin (70 nm) sections, which were mounted on nickel grids and processed for NT3 immunogold labeling. Ultrathin sections were treated with 1% periodic acid and 1% sodium periodate, washed with distilled water and TBS, and followed by blocking in 1% BSA for 30 min. After washing again with distilled water and TBS, samples were incubated overnight with anti-NT3 primary antibody (Table 2) at room temperature. Samples were washed in TBS, then permeabilized with a solution containing 1% BSA and 0.005% Tween-20 in tris(hydroxymethyl)aminomethane (pH = 7.6), followed by incubation with colloidal gold-conjugated anti-rabbit IgG secondary antibody (Table 2) for 3 h. Negative controls were performed to assess the specificity of the immunoreaction by omitting the primary antibody in the labeling protocol and incubating the sections only in the gold-conjugated secondary antibody (particle density was 0.047 ± 0.016 gold particles/µm^2^ in the negative control). Sections were counterstained with uranyl acetate (Merck, Darmstadt, Germany) and lead citrate (Leica Microsystems, Wetzlar, Germany) and were examined and photographed with a JEOL JEM 1400 transmission electron microscope (JEOL, Tokyo, Japan). The subcellular distributions of the gold particles labeling NT3 were determined in the myenteric ganglia. A total area of 5–10 myenteric ganglia per intestinal segment per condition was examined at a magnification of 20,000× with the TEM Center 1.6.9 software (JEOL, Tokyo, Japan). NT3 density was expressed as the total number of gold particles per unit area (μm^2^).

### 2.6. Statistical Analysis

Data were checked for Gaussian distribution by the Shapiro–Wilk normality test. Parametric tests were applied when the data met the assumptions of normality and homogeneity of variance. When these assumptions were not met, non-parametric tests were used. The specific statistical tests applied are indicated in the figure legends. All analyses were carried out with GraphPad Prism 11.0 (GraphPad Software, San Diego, CA, USA). A probability of *p* < 0.05 was set as the level of significance.

## 3. Results

### 3.1. Animal Models of Acute and Chronic Hyperglycemia

**Figure 1:**
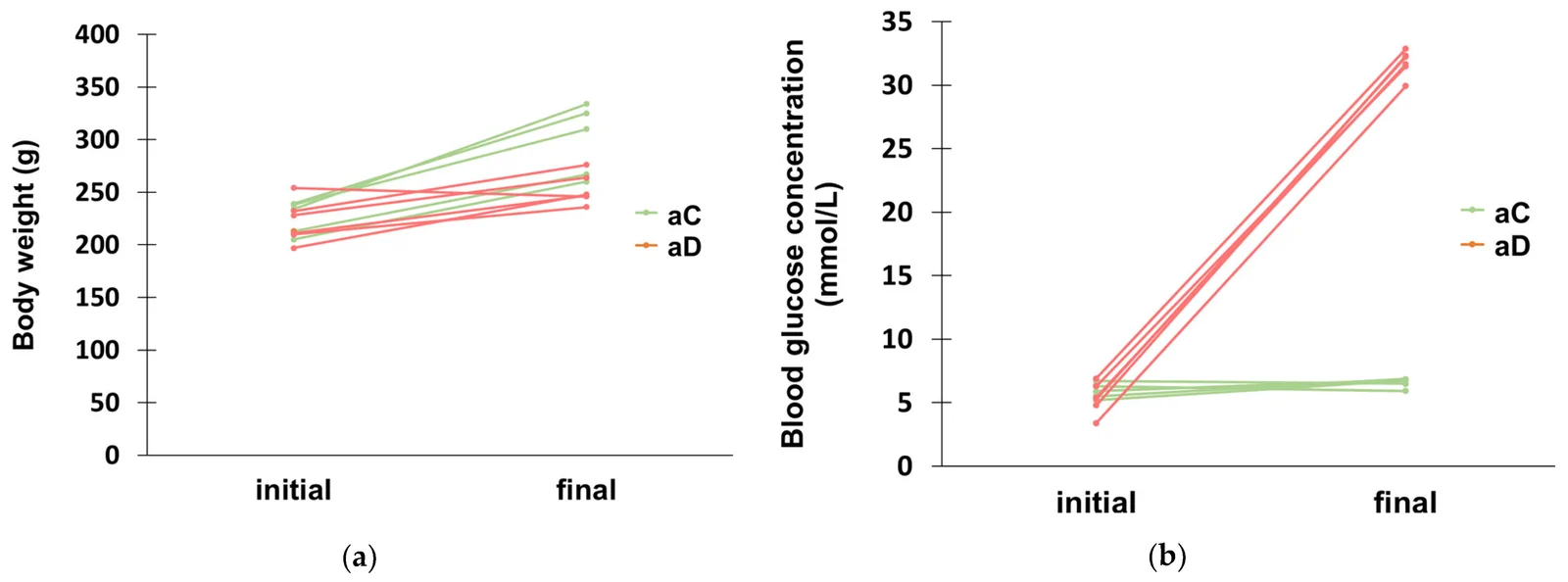
Initial and final body weight (**a**) and blood glucose concentration (**b**) of the acute experimental rats. aC = acute control (*n* = 5), aD = acute hyperglycemic (*n* = 6).

**Figure 2:**
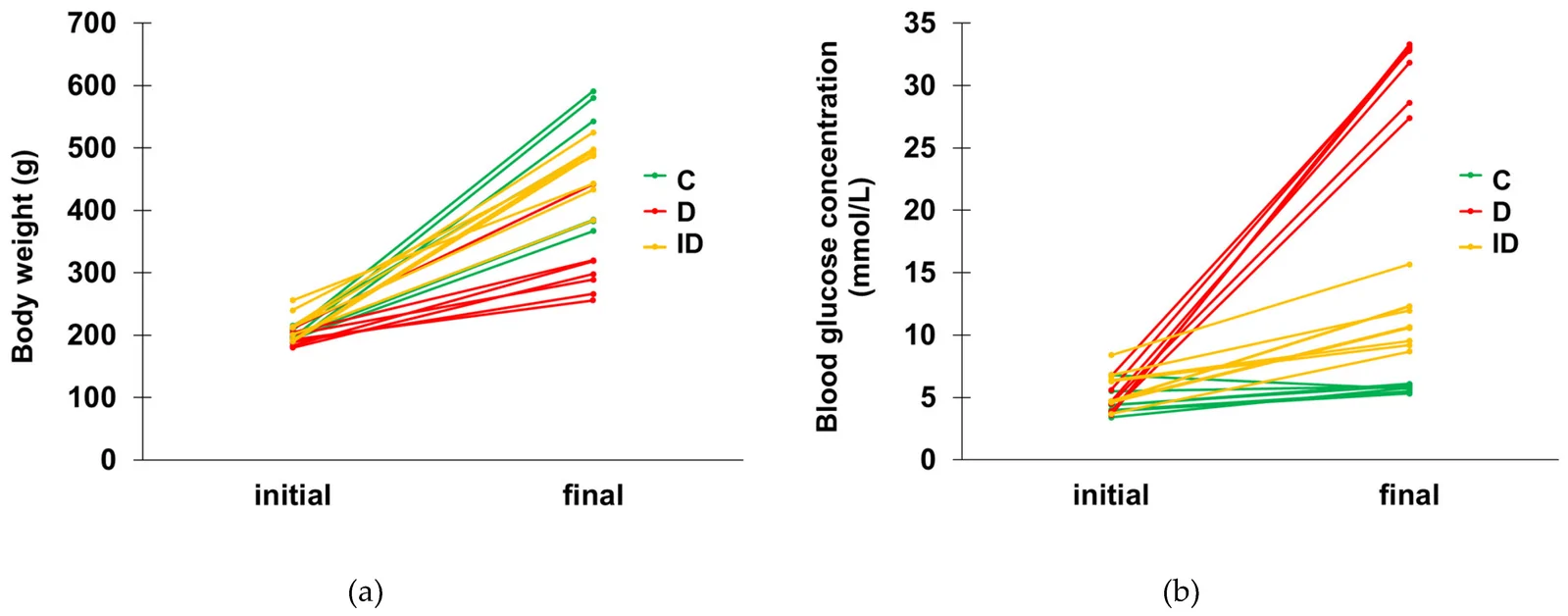
Initial and final body weight (**a**) or blood glucose concentration (**b**) in chronic experimental rats. C = control (*n* = 7), D = diabetic (*n* = 7), ID = insulin-treated diabetic (*n* = 8).

The initial and final body weight and glycemic changes in rats after a 1-week or 10-week experimental period are shown in Figure 1 and Figure 2, while changes in these parameters are represented in Table 3 and Table 4. The body weight of the rats increased in all experimental groups, although the body weight gain of hyperglycemic animals was significantly less than that of other groups (*p* < 0.01). The average blood glucose concentration of diabetic rats increased robustly (to more than 26 mmol/L) during the 1- or 10-week periods. Insulin treatment moderated the extremely high increase in blood glucose levels and resulted in a slight rise in the glucose concentrations of insulin-treated animals (5.4 ± 0.6 mmol/L) after their active nocturnal period and prior to daily insulin administration. No animal exclusions were made during the current experiments based on exclusion criteria.

### 3.2. Gut Region-Dependent Changes in the Proportion of GDNF–Immunoreactive Myenteric Neurons in Acute and Chronic Hyperglycemia

**Figure 3:**
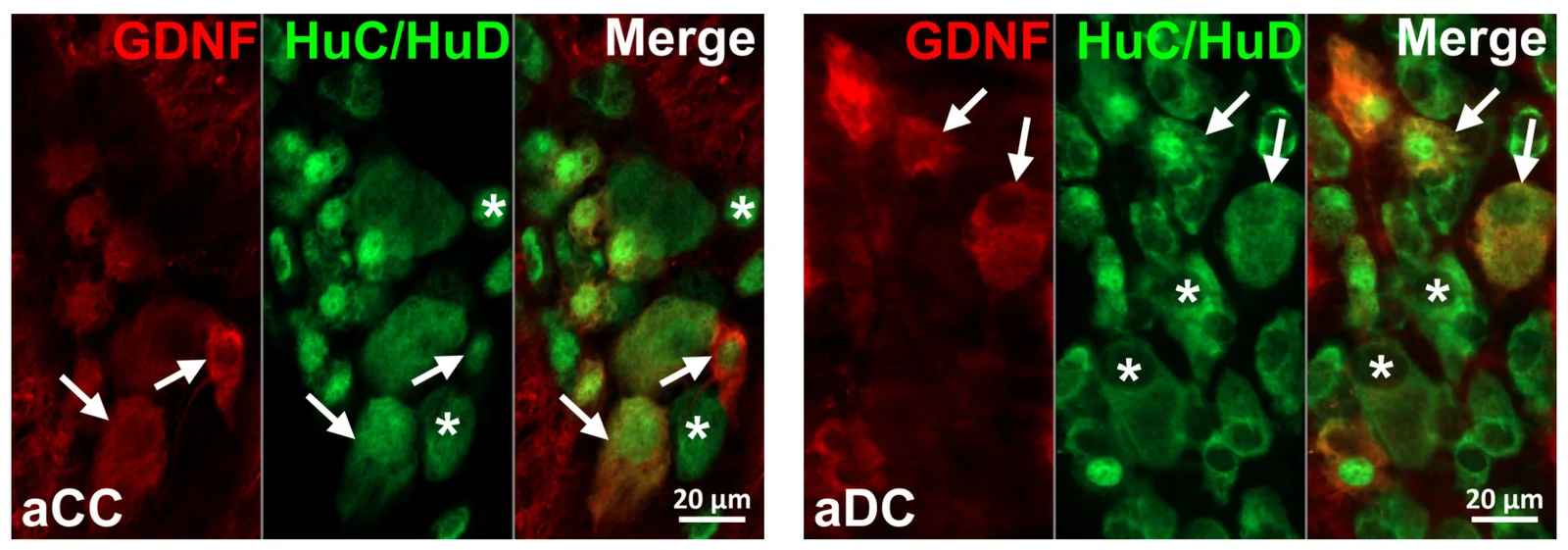
Representative fluorescent micrographs of whole-mount preparations of myenteric ganglia from the colon of control (aCC) and acute hyperglycemic (aDC) rats after glial cell line-derived neurotrophic factor (GDNF)-HuC/HuD double-labeling immunohistochemistry. HuC/HuD was used as a pan-neuronal marker to label myenteric neurons. Arrows: GDNF-immunoreactive myenteric neurons; stars: myenteric neurons; scale bars: 20 μm.

**Figure 4:**
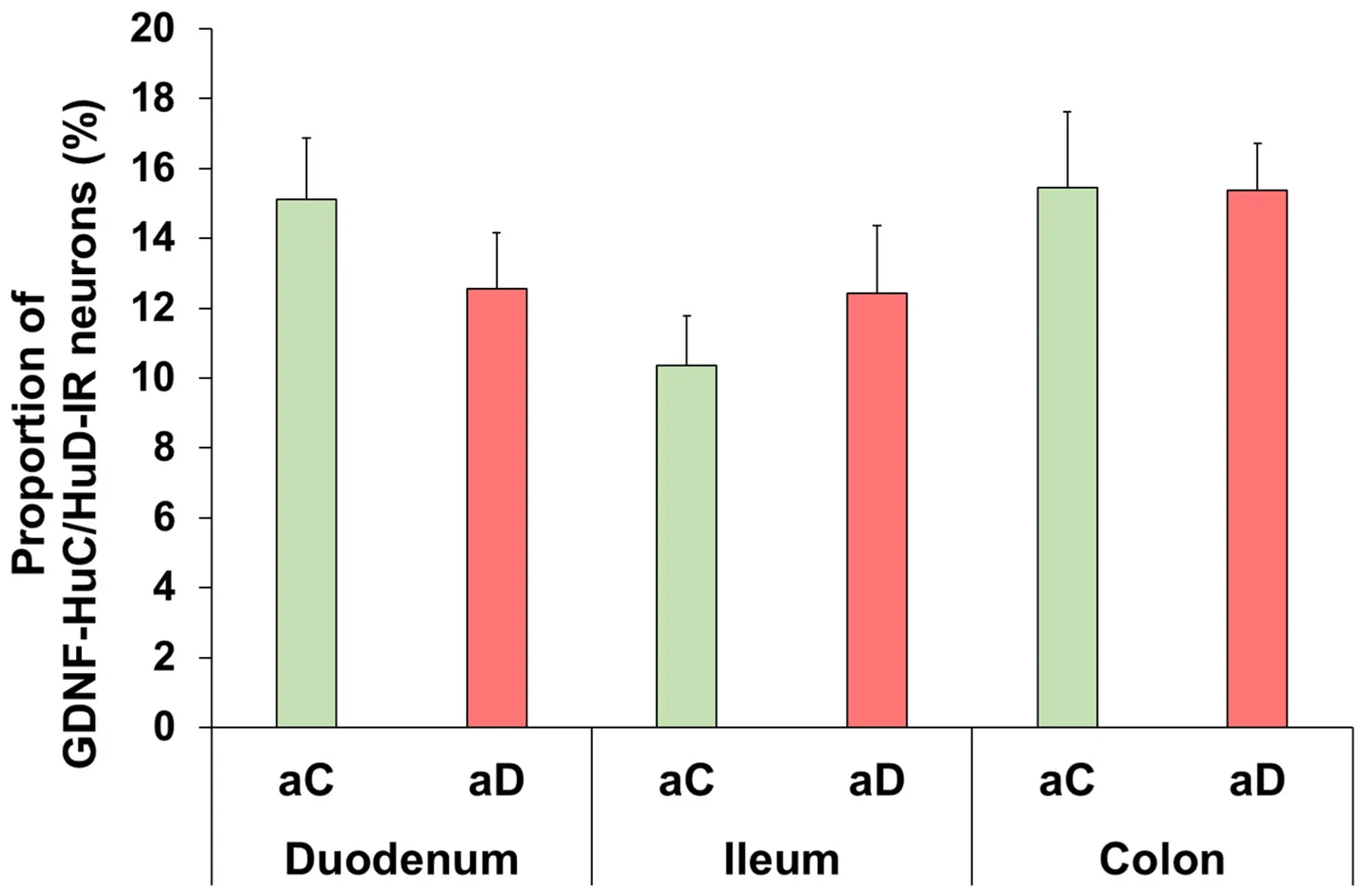
Proportion of glial cell line-derived neurotrophic factor (GDNF)–immunoreactive (IR) myenteric neurons per total neuronal number in different gut segments from control (aC) and acute hyperglycemic (aD) rats. Acute hyperglycemia did not induce significant changes in the proportion of GDNF-IR myenteric neurons in any investigated gut segments relative to controls. Statistics: Mann–Whitney test. Data are expressed as mean ± SEM. *n* = 30–50 myenteric ganglia/segment/experimental group from 3–4 animals.

**Figure 5:**
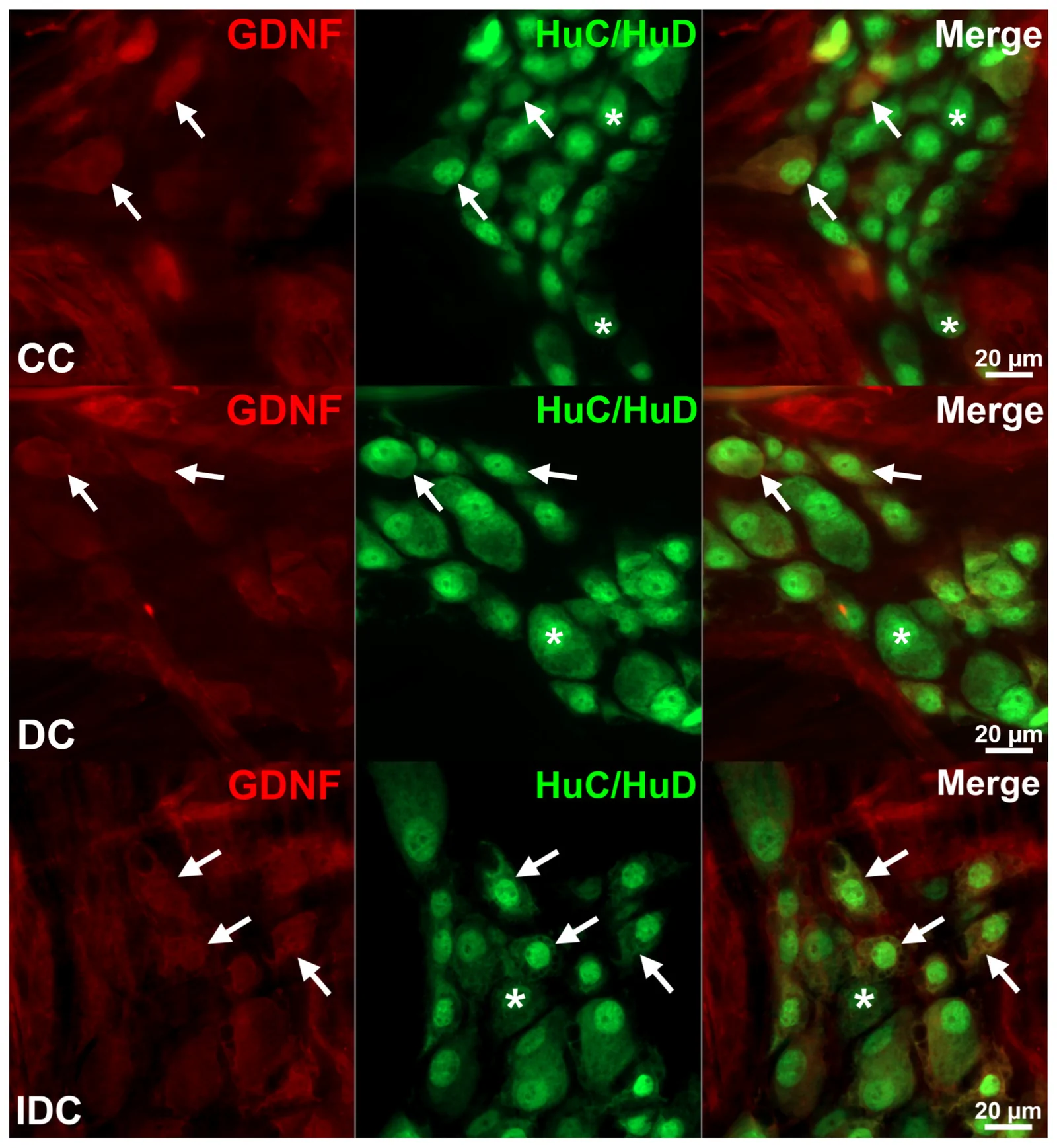
Representative fluorescent micrographs of whole-mount preparations of myenteric ganglia from the colon of control (CC), chronic diabetic (DC), and insulin-treated diabetic (IDC) rats after glial cell line-derived neurotrophic factor (GDNF)-HuC/HuD double-labeling immunohistochemistry. HuC/HuD was used as a pan-neuronal marker to label myenteric neurons. Arrows: GDNF–immunoreactive myenteric neurons, stars: myenteric neurons, scale bar: 20 μm.

**Figure 6:**
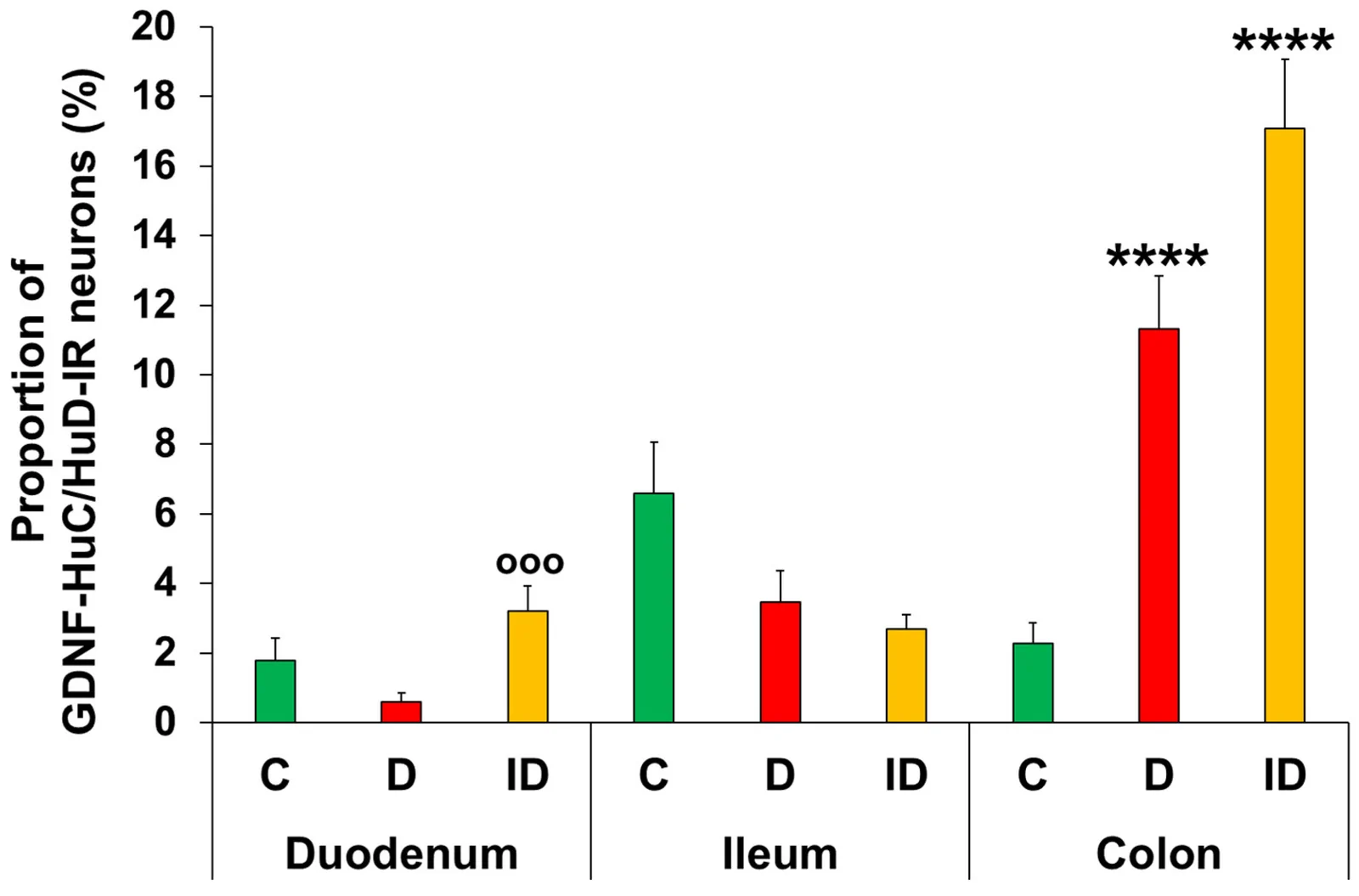
Proportion of glial cell line-derived neurotrophic factor (GDNF)–immunoreactive (IR) myenteric neurons per total neuronal number in different gut segments from control (C), chronic diabetic (D), and insulin-treated diabetic (ID) rats. Chronic hyperglycemia caused a significant increase in the proportion of colonic GDNF-IR myenteric neurons relative to controls. Statistics: Kruskal–Wallis test with Dunn’s multiple comparisons test. Data are expressed as mean ± SEM. **** *p* < 0.0001 (relative to controls); °°° *p* < 0.001 (between diabetic and insulin-treated diabetic groups). *n* = 30–50 myenteric ganglia/segment/experimental group from 3 animals.

To determine the proportion of GDNF–immunoreactive (IR) myenteric neurons, double-labeling fluorescent immunohistochemistry was applied (Figure 3, Figure 4, Figure 5 and Figure 6). After acute hyperglycemia, the proportion of GDNF-IR neurons per the total number of myenteric neurons did not show significant changes in any of the investigated gut segments compared to the controls (Figure 3 and Figure 4).

After chronic hyperglycemia, regionally distinct alterations of neuronal GDNF expression were observed in different gut segments of chronic diabetic rats (Figure 5 and Figure 6). In the small intestine (duodenum and ileum), the proportion of GDNF-IR neurons did not change significantly, while in the colon, it robustly increased relative to controls (11.33 ± 1.51% vs. 2.28 ± 0.59%, *p* < 0.0001). The effect of immediate insulin treatment was also segment-dependent; in the duodenum, the proportion of GDNF-IR neurons showed a 5-fold increase compared to the diabetics (3.2 ± 0.74% vs. 0.59 ± 0.25%, *p* < 0.001). In the ileum and colon, there was no significant difference between diabetic and insulin-treated diabetic groups.

### 3.3. Gut Segment-Dependent NT3 Expression in Intestinal Tissue Homogenates in Acute and Chronic Hyperglycemia

**Figure 7:**
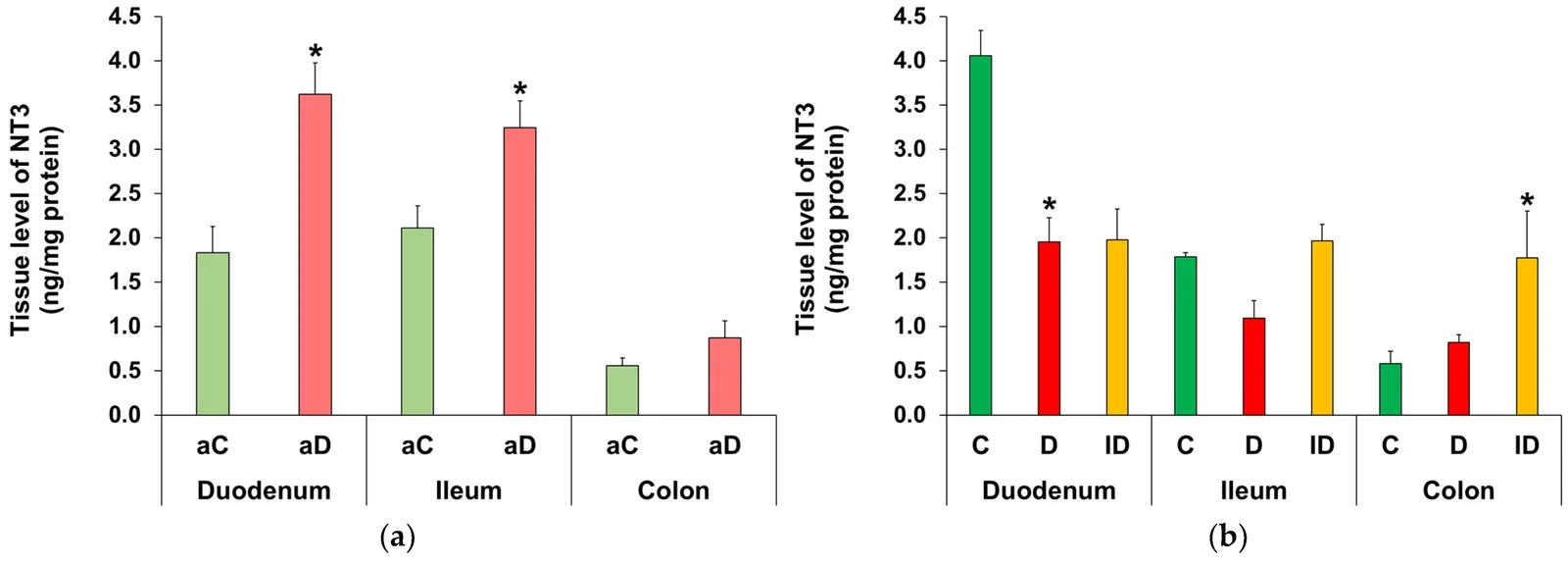
Tissue levels of neurotrophin-3 (NT3) in intestinal smooth muscle homogenates, including the myenteric plexus, from acute (**a**) and chronic (**b**) experiments. (**a**) The NT3 tissue level is significantly increased in the small intestinal segments in acute hyperglycemic rats compared to the controls. Statistics: Mann–Whitney test. Data are expressed as mean ± SEM. * *p* < 0.05 (relative to controls). aC: acute control, aD: acute hyperglycemic rats. *n* = 3–6 animals/segment per experimental group. (**b**) In the duodenum of chronic diabetic rats, the NT3 tissue level has halved compared to the controls. Statistics: Kruskal–Wallis test with Dunn’s multiple comparisons test. Data are expressed as mean ± SEM. * *p* < 0.05 (relative to controls). C: control, D: chronic diabetic, ID: insulin-treated diabetic rats. *n* = 3–5 animals/segment/experimental group.

Acute and chronic hyperglycemia induced intestinal segment-dependent alterations in the NT3 protein content of smooth muscle-myenteric plexus homogenates. In the acute hyperglycemic group, the expression of NT3 was significantly higher in the small intestinal segments compared to the controls (*p* < 0.05). Double the amount was measured in the duodenum (3.63 ± 0.35 ng/mg protein vs. 1.83 ± 0.29 ng/mg protein), while a 1.5-fold greater value was observed in the ileum (3.25 ± 0.3 ng/mg protein vs. 2.11 ± 0.25 ng/mg protein). In contrast, no significant difference was detected in the colon (Figure 7a).

In chronic diabetics, NT3 tissue levels of smooth muscle–myenteric plexus homogenates were decreased by 50% in the duodenum (1.96 ± 0.27 ng/mg protein vs. 4.06 ± 0.29 ng/mg protein, *p* < 0.05), while this level showed a decreasing tendency in the ileum (1.1 ± 0.2 ng/mg protein vs. 1.79 ± 0.04 ng/mg protein). In the colon, the 10-week hyperglycemia did not cause any changes in the NT3 tissue protein content. Insulin treatment did not prevent the diabetic changes in the duodenum, but resulted in a significant increase in NT3 content of colonic homogenates relative to control samples (Figure 7b).

Since we observed no changes in NT3 levels in the colonic tissue homogenates in either acute or chronic hyperglycemia, we focused on the small intestine in immunogold electron microscopy.

### 3.4. Gut Segment-Dependent Induction of NT3 in Myenteric Ganglia in Acute and Chronic Hyperglycemia

**Figure 8:**
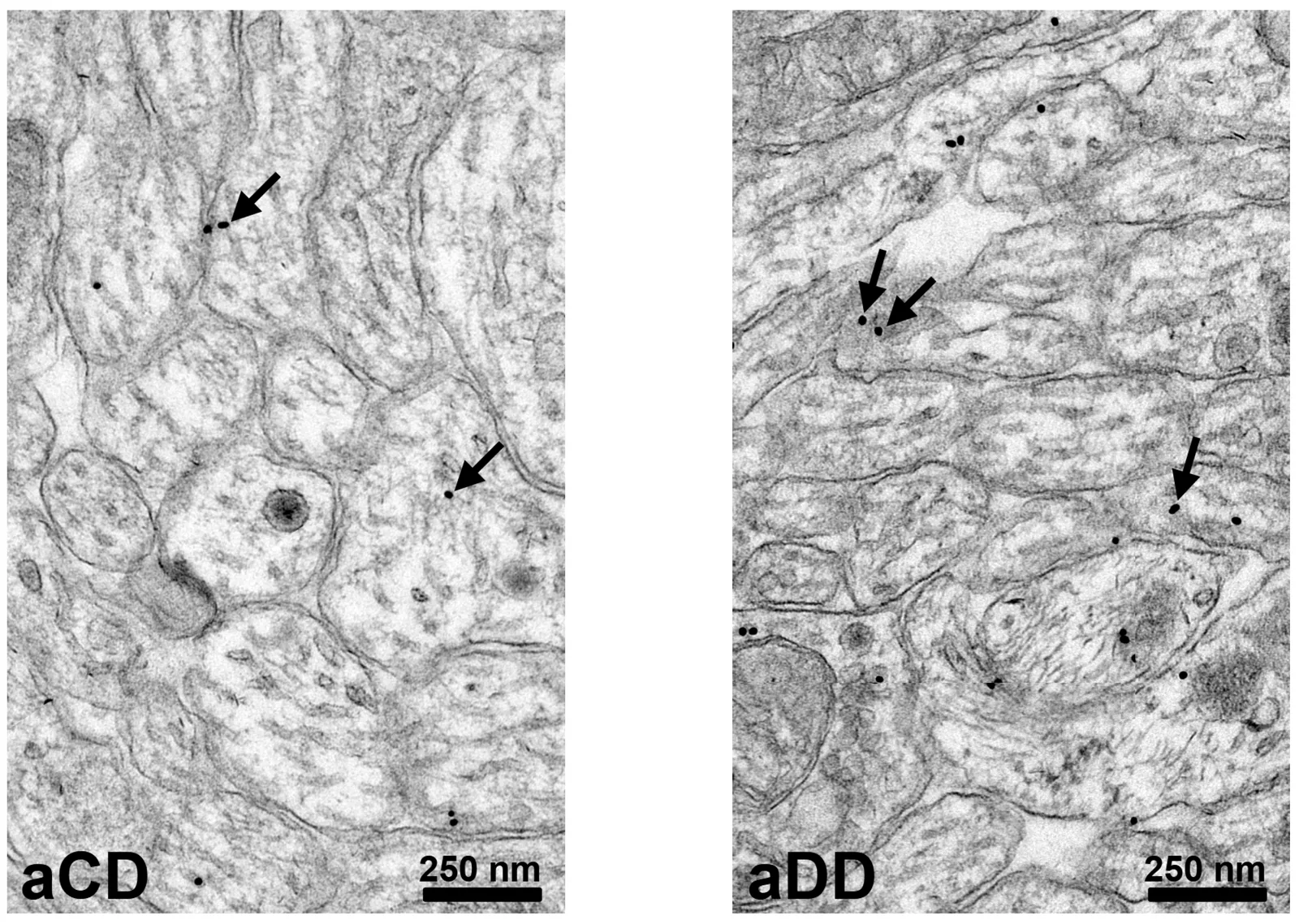
Representative electron micrographs of duodenal myenteric ganglia from control (aCD) and acutely hyperglycemic (aDD) rats after neurotrophin-3 (NT3) post-embedding immunohistochemistry. Arrows: 18 nm gold particles labeling NT3. Scale bars: 250 nm.

**Figure 9:**
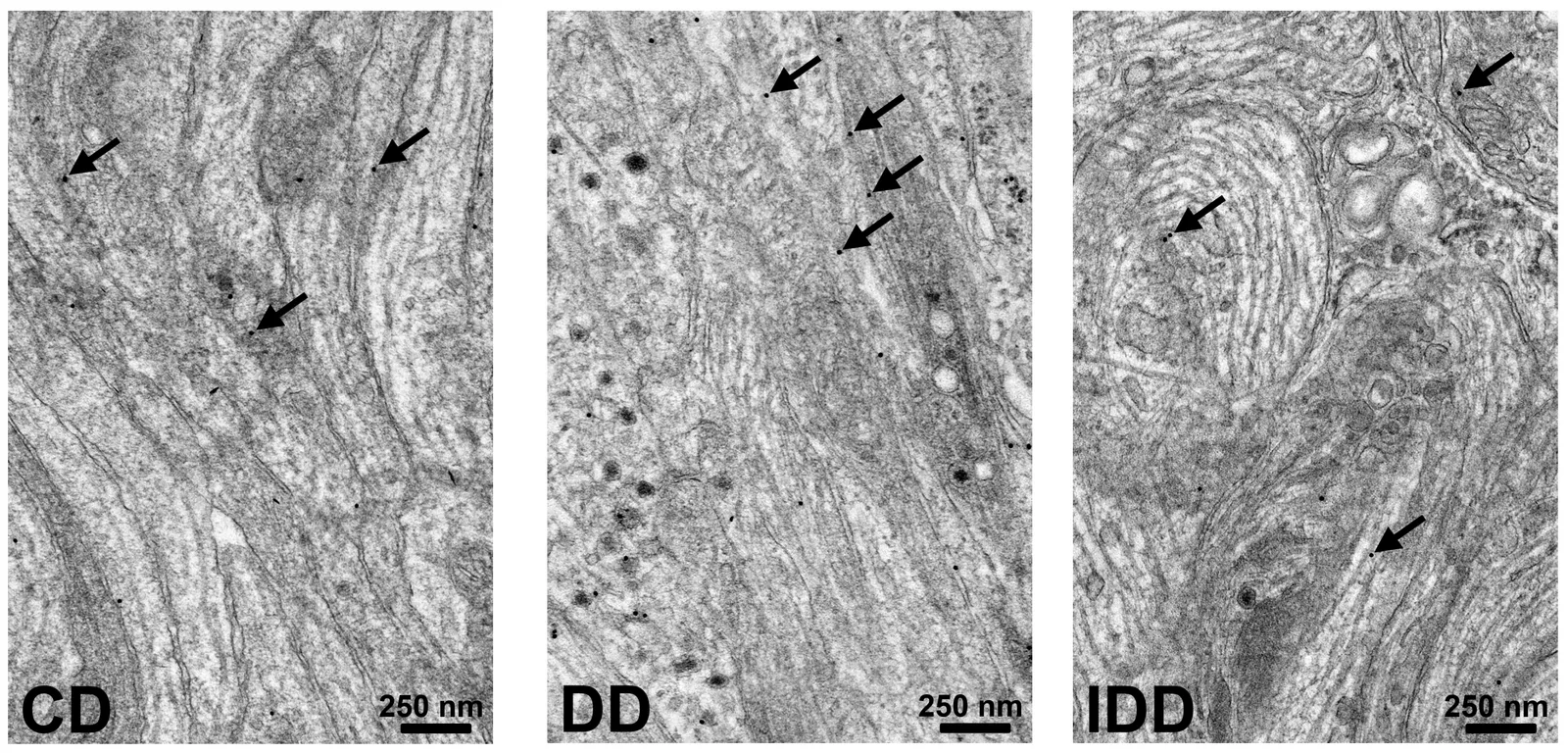
Representative electron micrographs of duodenal myenteric ganglia from control (CD), chronic diabetic (DD), and insulin-treated diabetic (IDD) rats after neurotrophin-3 (NT3) post-embedding immunohistochemistry. Arrows: 18 nm gold particles labeling NT-3. Scale bars: 250 nm.

**Figure 10:**
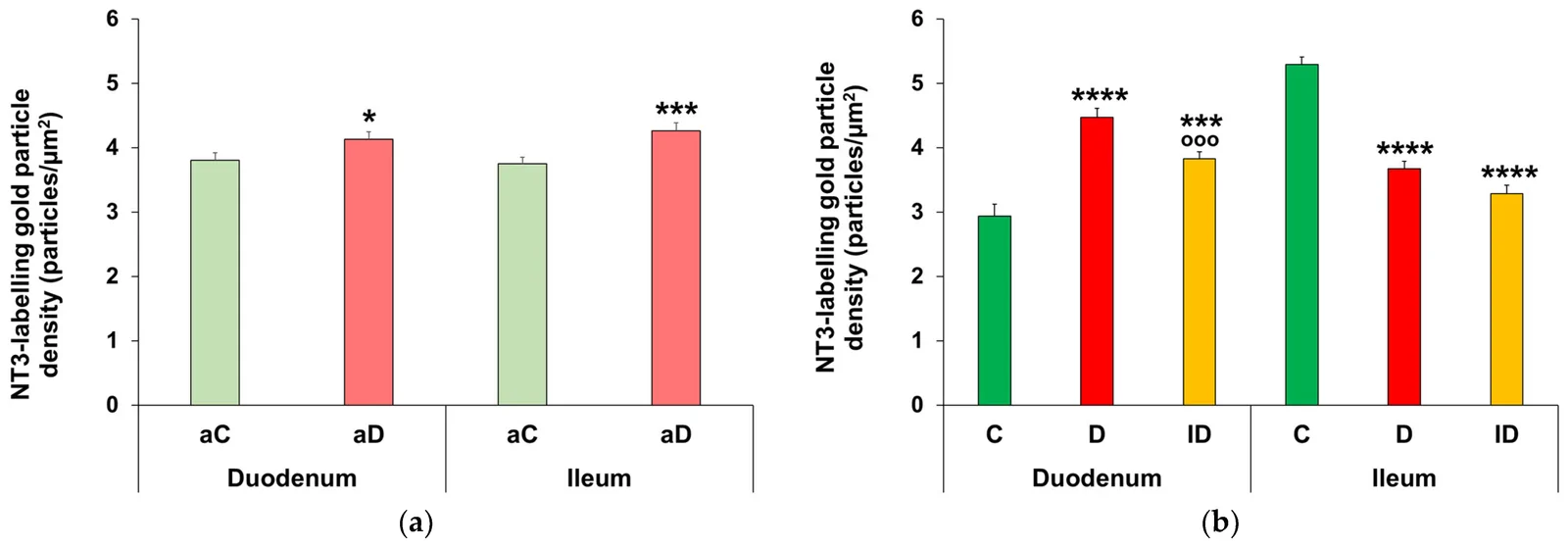
Quantitative evaluation of neurotrophin-3 (NT3)-labeled gold particles in the myenteric ganglia from acute (**a**) and chronic (**b**) experiments. (**a**) NT3 density significantly increased both in the duodenum and ileum of acute hyperglycemic rats relative to controls. Statistics: Mann–Whitney test. Data are expressed as mean ± SEM. * *p* < 0.05, *** *p* < 0.001 (relative to controls). aC: acute control, aD: acute hyperglycemic rats. *n* = 5–10 myenteric ganglia/segment/experimental group from 2 animals. (**b**) Duodenal NT3 density was significantly increased in chronic diabetic rats; however, it showed a marked decrease in the diabetic ileum compared to controls. Statistics: Kruskal–Wallis test with Dunn’s multiple comparisons test. Data are expressed as mean ± SEM. *** *p* < 0.001, **** *p* < 0.0001 (relative to controls); °°° *p* < 0.001 (between diabetics and insulin-treated diabetics). C: control, D: chronic diabetic, ID: insulin-treated diabetic rats. *n* = 5–10 myenteric ganglia/segment/experimental group from 2 to 4 animals.

The presence of NT3 was confirmed by immunogold electron microscopy in the myenteric ganglia (Figure 8, Figure 9 and Figure 10).

After acute hyperglycemia, the density of NT3-labeling gold particles significantly increased in both the duodenum and ileum relative to controls (duodenum: 4.13 ± 0.11 particles/μm^2^ vs. 3.8 ± 0.12 particles/μm^2^, *p* < 0.05; ileum: 4.27 ± 0.12 particles/μm^2^ vs. 3.75 ± 0.10 particles/μm^2^, *p* < 0.001; Figure 10a).

After chronic hyperglycemia, the number of duodenal NT3-labeling gold particles was markedly increased compared to the controls (4.48 ± 0.14 particle/μm^2^ vs. 2.94 ± 0.19 particle/μm^2^, *p* < 0.0001). In the insulin-treated diabetic group, it was significantly lower than in the diabetic group (*p* < 0.001), but still remained significantly higher than the control value (3.83 ± 0.11 particle/μm^2^, *p* < 0.001). In the diabetic ileum, the quantitative evaluation of gold particles labeling NT3 showed a significant decrease relative to the controls (3.68 ± 0.12 particle/μm^2^ vs. 5.29 ± 0.12 particle/μm^2^, *p* < 0.0001). In the insulin-treated diabetic rats, it did not change relative to the diabetic group in this gut segment (Figure 10b).

## 4. Discussion

In the present study, we examined the levels of two neurotrophic factors, which continue to be expressed in the intestine throughout adulthood. Gut-segment-specific changes in GDNF and NT3 expression in myenteric ganglia were investigated in short- and long-term hyperglycemia. Since the examination of different intestinal segments from humans is not feasible, we used the same STZ rat model as the one in which intestinal region-dependent enteric neuropathy and damage in gut motility have also been described in untreated and insulin-treated diabetic groups and which has clinical importance [3].

The ratio of GDNF-IR myenteric neurons to total neurons was calculated using GDNF-HuC/HuD double-labeling on whole-mount preparations from the duodenum, ileum and colon. No changes were detected in any of the investigated gut segments in the acute hyperglycemia group. However, chronic diabetes induced a marked increase in the colon, which was further elevated by insulin treatment. Myenteric neurons may take up GDNF secreted by enteric glial cells [18,19,40], intestinal smooth muscle cells [15,16] and enterocytes [17]. We rely on the manufacturer’s statement that the anti-GDNF antibody used detects mature GDNF protein only, and not the precursor form (proGDNF). Earlier studies demonstrated that the main source of GDNF is the smooth muscle layers of the gut wall, but its receptors, the RET receptor tyrosine kinase and GFRα1, are mainly expressed in myenteric ganglia [16]. According to the neurotrophic factor concept, the neurotrophic factor produced by the target tissue protects the innervating neurons selectively. GDNF is also able to induce the mRNA expression of its receptors [16]. Wright et al. [41] described the characteristic GDNF receptors of glial cells and different neuronal subtypes in the mouse distal colon. In STZ-induced chronic diabetes, decreased myenteric neuronal number and unchanged glial density were measured in the colonic myenteric plexus [39], which was accompanied by faster distal colonic transit time compared to control rats in the same animal model [3]. The increased proportion of GDNF-containing neurons may be a defense mechanism in the pro-oxidant colonic environment, although it could not prevent cell death [42]. Additionally, the protective role of GDNF was demonstrated in another diabetic animal model. The diabetic loss of neuronal nitric oxide synthase-containing neurons and total neuronal number in the wild-type mouse ileum was ameliorated in GDNF-overexpressing transgenic mice [21]. Moreover, in the aging gut, GDNF increased the expression of the antioxidant Cu/Zn superoxide dismutase and catalase in cultured enteric ganglion cells [43].

The NT3 protein content of tissue homogenates dominated by intestinal smooth muscle layers was measured from the duodenum, ileum, and colon after acute and chronic hyperglycemia to evaluate the segment-specific changes in short-term and long-lasting hyperglycemia. Luo et al. [22] previously reported similar results, stating that acute hyperglycemia significantly increases the NT3 protein content of the ileum, and long-lasting hyperglycemia does not cause significant differences. Our study confirmed that the observed changes are segment-specific. In the duodenum, the NT3 protein level was doubled after acute hyperglycemia but halved in chronic diabetes. Even though the proportion of GDNF-IR neurons has changed in the colon, we did not find any differences in the tissue level of NT3 in this gut segment between acute and chronic hyperglycemic rats and control rats.

To study the NT3 content in small intestinal myenteric ganglia, immunogold electron microscopy was used. A limitation of the study is the lack of discrimination between the precursor and the mature form of NT3. An increased density of NT3-labeling gold particles was detected in duodenal myenteric ganglia both in acute and chronic hyperglycemia, and the difference was even more pronounced after chronic diabetes. In the ileal ganglia, the acutely increased NT3 content significantly decreased during the chronic experiment. Earlier studies reported that in chronic diabetes, the neuronal density did not change in duodenal myenteric ganglia, but it decreased in ileal myenteric ganglia [39], which was accompanied by accelerated small intestinal transit time compared to controls in the same experimental conditions [3]. These results suggest that the unchanged neuronal number in the duodenal myenteric plexus may be maintained via increased NT3 levels during chronic hyperglycemia. Prior research described that NT3 protects neurons. Pre-ischemic exercise reduced cell death in the hippocampal CA1 region through increasing the gene expression and protein level of NT3, NT4, TrkB, and TrkC [44]. Pre-treatment with NT3 decreased cell death induced by hydrogen peroxide exposure in a culture of enteric ganglion cells [45]. It should be noted that hyperglycemia-induced changes in NT3 density were partially prevented in the duodenal ganglia of the insulin-treated group; however, insulin treatment was not effective in the case of other investigated parameters and gut segments. This confirms that insulin itself does not provide complete protection against diabetic changes even under appropriate glycemic control [3].

The possible diagnostic or therapeutic applications of GDNF and NT3 have been investigated intensively in neurodegenerative diseases. GDNF is a possible therapeutic factor in Parkinson’s disease [46,47] through its regulatory role in neuroinflammation [48]. A newly developed recombinant NT3 protein is an innovative therapeutic direction in the treatment of Alzheimer’s disease, because it alleviated hippocampal neurotoxicity by decreasing inflammation and oxidative stress [49].

In conclusion, our results suggest that neurotrophic factors promote the adaptation of myenteric neurons to long-lasting hyperglycemia in a segment-specific manner. The expression of NT3 was elevated in the duodenal myenteric ganglia, while the proportion of GDNF-IR myenteric neurons was increased in the colon. The expression changes in these neurotrophic factors depend on the duration of hyperglycemia. Based on these segment-specific and time-dependent expression alterations, we propose that, in the duodenum, the increased NT3 content may support cell survival. In the colon, which is more vulnerable to diabetes-induced oxidative stress, the elevated GDNF level may be a defense mechanism despite the decrease in the total neuronal number.

The following are the limitations of our study. Only STZ-induced diabetic models were used, with only male rats, and a small sample size in each group. The insulin doses lasted a few days; therefore, the insulin-treated group was not included in the acute experiments. Based on control-value discrepancies, these could stem from age; therefore, data from the acute and chronic experiments were not directly compared. Further investigations are needed to verify successful signal transduction by which neurotrophic factors are able to exert neuroprotective roles, such as measuring corresponding receptors of GDNF, such as phosphorylated RET and GFRα1, in myenteric ganglia. Moreover, the measurement of GDNF mRNA needs to test neuronal *de novo* synthesis of GDNF in myenteric ganglia. The pro- versus mature forms of neurotrophins were not discriminated. These additions are important, especially since these molecules may be used as therapeutic applications in the treatment of gastrointestinal side effects in diabetic patients.

## Figures and Tables

**Table 1:** Primary and secondary antibodies and working dilutions applied in the fluorescence experiments.

**Primary antibody**	**Host**	**Final dilution**	**Product code**	**Company**
anti-GDNF monoclonal	mouse	1:100	sc-13147	Santa Cruz Biotechnology, Inc., Dallas, TX, USA
anti-HuC/HuD monoclonal	rabbit	1:200	ab184267	Abcam, Cambridge, UK
**Secondary antibody**	**Host**	**Final dilution**	**Product code**	**Company**
anti-mouse Cy^TM^3	goat	1:200	115-165-003	Jackson ImmunoResearch Laboratories, West Grove, PA, USA
anti-rabbit Alexa Fluor^®^ 488	goat	1:200	A11008	Invitrogen, Waltham, MA, USA

**Table 2:** Primary and secondary antibodies and working dilutions used in the electron microscopy experiments.

**Primary antibody**	**Host**	**Final dilution**	**Product code**	**Company**
anti-NT3 polyclonal	rabbit	1:50	18084-1-AP	Proteintech, Rosemont, IL, USA
**Secondary antibody**	**Host**	**Final dilution**	**Product code**	**Company**
anti-rabbit IgG conjugated to 18 nm gold particles	goat	1:20	111-215-144	Jackson ImmunoResearch Laboratories, West Grove, PA, USA

**Table 3:** Changes in body weight and blood glucose concentrations of the acute experimental groups. aC = acute control, aD = acute hyperglycemic.

	Changes in Body Weight (g)	Changes in Blood Glucose Concentration (mmol/L)
aC (*n* = 5)	73.4 ± 8.9	0.64 ± 0.4
aD (*n* = 6)	30.8 ± 8.5 **	26.4 ± 0.33 ****

Data are expressed as mean ± SEM; ** *p* = 0.0074, **** *p* < 0.0001. Data were analyzed by Welch’s test.

**Table 4:** Changes in body weight and blood glucose characteristics in the chronic experimental groups.

	Changes in Body Weight (g)	Changes in Blood Glucose Concentration (mmol/L)
C (*n* = 7)	278.4 ± 36.7	1.12 ± 0.43
D (*n* = 7)	118.4 ± 21.1 **	26.7 ± 0.88 ****
ID (*n* = 8)	254.5 ± 18.6 °°	5.4 ± 0.6 ***^,^°°°°

Data are expressed as mean ± SEM; ** *p* < 0.01, *** *p* < 0.001, **** *p* < 0.0001 vs. C, and °° *p* < 0.01, °°°° *p* < 0.0001 vs. D. Data were analyzed by the Kruskal–Wallis test with Dunn’s multiple comparisons test.

## Data Availability

The dataset is available from the corresponding author at bmarcsi@bio.u-szeged.hu e-mail address.

## Abbreviations

The following abbreviations are used in this manuscript:

aC	acute control
aCC	acute control colon
aCD	acute control duodenum
aCI	acute control ileum
aD	acute hyperglycemic
aDC	acute hyperglycemic colon
aDD	acute hyperglycemic duodenum
aDI	acute hyperglycemic ileum
BSA	bovine serum albumin
CC	chronic control colon
CD	chronic control duodenum
CI	chronic control ileum
DC	chronic diabetic colon
DD	chronic diabetic duodenum
DI	chronic diabetic ileum
ELISA	enzyme-linked immunosorbent assay
ENS	enteric nervous system
GDNF	glial cell line-derived neurotrophic factor
GFRα1	GDNF family receptor alpha 1
IDC	insulin-treated diabetic colon
IDD	insulin-treated diabetic duodenum
IDI	insulin-treated diabetic ileum
IR	immunoreactive
NT3	neurotrophin-3
p75^NTR^	p75 neurotrophin receptor
PB	phosphate buffer
PBS	phosphate-buffered saline
proGDNF	precursor form of glial cell line-derived neurotrophic factor
proNT3	precursor form of neurotrophin-3
RET	rearranged during transfection
STZ	streptozotocin
TBS	tris(hydroxymethyl)aminomethane-buffered saline
Trk	tropomyosin receptor kinase

## References

[1] Uranga-Ocio J.A., Bastús-Díez S., Delkáder-Palacios D., García-Cristóbal N., Leal-García M.Á., Abalo-Delgado R. (2015). Enteric neuropathy associated to diabetes mellitus. Rev. Esp. Enferm. Dig..

[2] Morarach K., Mikhailova A., Knoflach V., Memic F., Kumar R., Li W., Ernfors P., Marklund U. (2021). Diversification of molecularly defined myenteric neuron classes revealed by single-cell RNA sequencing. Nat. Neurosci..

[3] Izbéki F., Wittman T., Rosztóczy A., Linke N., Bódi N., Fekete E., Bagyánszki M. (2008). Immediate insulin treatment prevents gut motility alterations and loss of nitrergic neurons in the ileum and colon of rats with streptozotocin-induced diabetes. Diabetes Res. Clin. Pract..

[4] Bagyánszki M., Bódi N. (2023). Key elements determining the intestinal region-specific environment of enteric neurons in type 1 diabetes. World J. Gastroenterol..

[5] Yao L., Zhang D., Bernd P. (1994). The onset of neurotrophin and trk mRNA expression in early embryonic tissues of the quail. Dev. Biol..

[6] Moore M.W., Klein R.D., Fariñas I., Sauer H., Armanini M., Phillips H., Reichardt L.F., Ryan A.M., Carver-Moore K., Rosenthal A. (1996). Renal and neuronal abnormalities in mice lacking GDNF. Nature.

[7] Chalazonitis A., Pham T.D., Rothman T.P., DiStefano P.S., Bothwell M., Blair-Flynn J., Tessarollo L., Gershon M.D. (2001). Neurotrophin-3 is required for the survival-differentiation of subsets of developing enteric neurons. J. Neurosci. Off. J. Soc. Neurosci..

[8] Rodrigues D.M., Li A.Y., Nair D.G., Blennerhassett M.G. (2011). Glial cell line-derived neurotrophic factor is a key neurotrophin in the postnatal enteric nervous system. Neurogastroenterol. Motil..

[9] Airaksinen M.S., Saarma M. (2002). The GDNF family: Signalling, biological functions and therapeutic value. Nat. Rev. Neurosci..

[10] Mol P., Balaya R.D.A., Dagamajalu S., Babu S., Chandrasekaran P., Raghavan R., Suresh S., Ravishankara N., Raju A.H., Nair B. (2023). A network map of GDNF/RET signaling pathway in physiological and pathological conditions. J. Cell Commun. Signal..

[11] Virtanen H.T., Choopanian P., Porokuokka L.L., Forsgård R., Garton D.R., Olfat S., Korpela R., Mirzaie M., Andressoo J.-O. (2024). Interindividual Variation in Gut Nitrergic Neuron Density Is Regulated By GDNF Levels and ETV1. Cell. Mol. Gastroenterol. Hepatol..

[12] Lonka-Nevalaita L., Lume M., Leppänen S., Jokitalo E., Peränen J., Saarma M. (2010). Characterization of the intracellular localization, processing, and secretion of two glial cell line-derived neurotrophic factor splice isoforms. J. Neurosci. Off. J. Soc. Neurosci..

[13] Ubhi K., Rockenstein E., Mante M., Inglis C., Adame A., Patrick C., Whitney K., Masliah E. (2010). Neurodegeneration in a transgenic mouse model of multiple system atrophy is associated with altered expression of oligodendroglial-derived neurotrophic factors. J. Neurosci. Off. J. Soc. Neurosci..

[14] Duarte Azevedo M., Sander S., Tenenbaum L. (2020). GDNF, A Neuron-Derived Factor Upregulated in Glial Cells during Disease. J. Clin. Med..

[15] Han T.Y., Lourenssen S., Miller K.G., Blennerhassett M.G. (2015). Intestinal smooth muscle phenotype determines enteric neuronal survival via GDNF expression. Neuroscience.

[16] Böttner M., Barrenschee M., Hellwig I., Harde J., Egberts J.-H., Becker T., Zorenkov D., Schäfer K.-H., Wedel T. (2013). The GDNF System Is Altered in Diverticular Disease—Implications for Pathogenesis. PLoS ONE.

[17] Meir M., Flemming S., Burkard N., Bergauer L., Metzger M., Germer C.-T., Schlegel N. (2015). Glial cell line-derived neurotrophic factor promotes barrier maturation and wound healing in intestinal epithelial cells in vitro. Am. J. Physiol. Gastrointest. Liver Physiol..

[18] von Boyen G.B.T., Steinkamp M., Geerling I., Reinshagen M., Schäfer K.H., Adler G., Kirsch J. (2006). Proinflammatory cytokines induce neurotrophic factor expression in enteric glia: A key to the regulation of epithelial apoptosis in Crohn’s disease. Inflamm. Bowel Dis..

[19] von Boyen G.B.T., Schulte N., Pflüger C., Spaniol U., Hartmann C., Steinkamp M. (2011). Distribution of enteric glia and GDNF during gut inflammation. BMC Gastroenterol..

[20] Xiao W., Wang W., Chen W., Sun L., Li X., Zhang C., Yang H. (2014). GDNF is involved in the barrier-inducing effect of enteric glial cells on intestinal epithelial cells under acute ischemia reperfusion stimulation. Mol. Neurobiol..

[21] Anitha M., Gondha C., Sutliff R., Parsadanian A., Mwangi S., Sitaraman S.V., Srinivasan S. (2006). GDNF rescues hyperglycemia-induced diabetic enteric neuropathy through activation of the PI3K/Akt pathway. J. Clin. Investig..

[22] Luo P., Liu D., Li C., He W.-X., Zhang C.-L., Chang M.-J. (2018). Enteric glial cell activation protects enteric neurons from damage due to diabetes in part via the promotion of neurotrophic factor release. Neurogastroenterol. Motil..

[23] Heymach J.V., Shooter E.M. (1995). The biosynthesis of neurotrophin heterodimers by transfected mammalian cells. J. Biol. Chem..

[24] Hempstead B.L. (2006). Dissecting the diverse actions of pro- and mature neurotrophins. Curr. Alzheimer Res..

[25] Teng K.K., Felice S., Kim T., Hempstead B.L. (2010). Understanding Proneurotrophin Actions: Recent Advances and Challenges. Dev. Neurobiol..

[26] Shang H., Meng X., Wang B., Feng X., Ma R., Hu H., Bekker A., Tao Y.-X. (2026). Contribution of proneurotrophin-3 to nerve trauma-induced neuropathic pain through promoting TrkC-mediated increase of CCL2 in primary sensory neurons. Brain. Behav. Immun..

[27] Sharma D., Feng X., Wang B., Shang H., Yasin B., Bekker A., Hu H., Tao Y.-X. (2025). Proneurotrophin-3 contributes to chemotherapy-induced neuropathic pain through TrkC-mediated CCL2 elevation in DRG neurons. EMBO Rep..

[28] Chalazonitis A. (2004). Neurotrophin-3 in the development of the enteric nervous system. Prog. Brain Res..

[29] Reichardt L.F. (2006). Neurotrophin-regulated signalling pathways. Philos. Trans. R. Soc. Lond. B Biol. Sci..

[30] Bothwell M. (2016). Recent advances in understanding neurotrophin signaling. F1000Research.

[31] Al-Qudah M., Shammala D.A., Al-Dwairi A., Al-Shboul O., Mustafa A.G. (2017). Dextran sodium sulphate (DSS)-induced colitis alters the expression of neurotrophins in smooth muscle cells of rat colon. Physiol. Res..

[32] Lucini C., Maruccio L., de Girolamo P., Vega J.A., Castaldo L. (2002). Localisation of neurotrophin—Containing cells in higher vertebrate intestine. Anat. Embryol..

[33] Kennedy A.J., Wellmer A., Facer P., Saldanha G., Kopelman P., Lindsay R.M., Anand P. (1998). Neurotrophin-3 is increased in skin in human diabetic neuropathy. J. Neurol. Neurosurg. Psychiatry.

[34] Huang T.-J., Sayers N.M., Verkhratsky A., Fernyhough P. (2005). Neurotrophin-3 prevents mitochondrial dysfunction in sensory neurons of streptozotocin-diabetic rats. Exp. Neurol..

[35] Christianson J.A., Ryals J.M., Johnson M.S., Dobrowsky R.T., Wright D.E. (2007). Neurotrophic modulation of myelinated cutaneous innervation and mechanical sensory loss in diabetic mice. Neuroscience.

[36] Wirth R., Bódi N., Maróti G., Bagyánszki M., Talapka P., Fekete É., Bagi Z., Kovács K.L. (2014). Regionally distinct alterations in the composition of the gut microbiota in rats with streptozotocin-induced diabetes. PLoS ONE.

[37] Arora T., Vanslette A.M., Hjorth S.A., Bäckhed F. (2021). Microbial regulation of enteroendocrine cells. Med.

[38] Brown H., Esterházy D. (2021). Intestinal immune compartmentalization: Implications of tissue specific determinants in health and disease. Mucosal Immunol..

[39] Onhausz B., Barta B.P., Egyed-Kolumbán A., Szalai Z., Bagyánszki M., Bódi N. (2026). Enteric Glial Network in Diabetes: Quantitative Changes of Glial Density in Rats in Response to Acute and Chronic Hyperglycaemia. Biomedicines.

[40] Meir M., Kannapin F., Diefenbacher M., Ghoreishi Y., Kollmann C., Flemming S., Germer C.-T., Waschke J., Leven P., Schneider R. (2021). Intestinal Epithelial Barrier Maturation by Enteric Glial Cells Is GDNF-Dependent. Int. J. Mol. Sci..

[41] Wright C.M., Schneider S., Smith-Edwards K.M., Mafra F., Leembruggen A.J.L., Gonzalez M.V., Kothakapa D.R., Anderson J.B., Maguire B.A., Gao T. (2021). scRNA-Seq Reveals New Enteric Nervous System Roles for GDNF, NRTN, and TBX3. Cell. Mol. Gastroenterol. Hepatol..

[42] Jancsó Z., Bódi N., Borsos B., Fekete É., Hermesz E. (2015). Gut region-specific accumulation of reactive oxygen species leads to regionally distinct activation of antioxidant and apoptotic marker molecules in rats with STZ-induced diabetes. Int. J. Biochem. Cell Biol..

[43] Korsak K., Dolatshad N.F., Silva A.T., Saffrey M.J. (2012). Ageing of enteric neurons: Oxidative stress, neurotrophic factors and antioxidant enzymes. Chem. Cent. J..

[44] Moghanlou A.E., Yazdanian M., Roshani S., Demirli A., Seydyousefi M., Metz G.A.S., Faghfoori Z. (2023). Neuroprotective effects of pre-ischemic exercise are linked to expression of NT-3/NT-4 and TrkB/TrkC in rats. Brain Res. Bull..

[45] Korsak K., Silva A.T., Saffrey M.J. (2012). Differing effects of NT-3 and GDNF on dissociated enteric ganglion cells exposed to hydrogen peroxide in vitro. Neurosci. Lett..

[46] Alster P., Otto-Ślusarczyk D., Szlufik S., Duszyńska-Wąs K., Drzewińska A., Wiercińska-Drapało A., Struga M., Kutyłowski M., Friedman A., Madetko-Alster N. (2024). The significance of glial cell line-derived neurotrophic factor analysis in Progressive Supranuclear Palsy. Sci. Rep..

[47] Runeberg-Roos P., Penn R.D. (2020). Improving therapeutic potential of GDNF family ligands. Cell Tissue Res..

[48] Singh G., Sikder A., Phatale V., Srivastava S., Singh S.B., Khatri D.K. (2023). Therapeutic potential of GDNF in neuroinflammation: Targeted delivery approaches for precision treatment in neurological diseases. J. Drug Deliv. Sci. Technol..

[49] Ding X., Zuo Y., Liu Z., Sun Y., Wang L., Xie Y., Liu G., Liu C. (2025). Recombinant neurotrophin-3 with the ability to penetrate the blood-brain barrier: A new strategy against Alzheimer’s disease. Int. J. Biol. Macromol..

